# Supplementary figures and images for: Genome-wide analysis of circular RNAs in goat skin fibroblast cells in response to Orf virus infection
Source: PeerJ. 2019 Jan 22;7:e6267. doi: 10.7717/peerj.6267 (PMC6346991; doi:10.7717/peerj.6267)

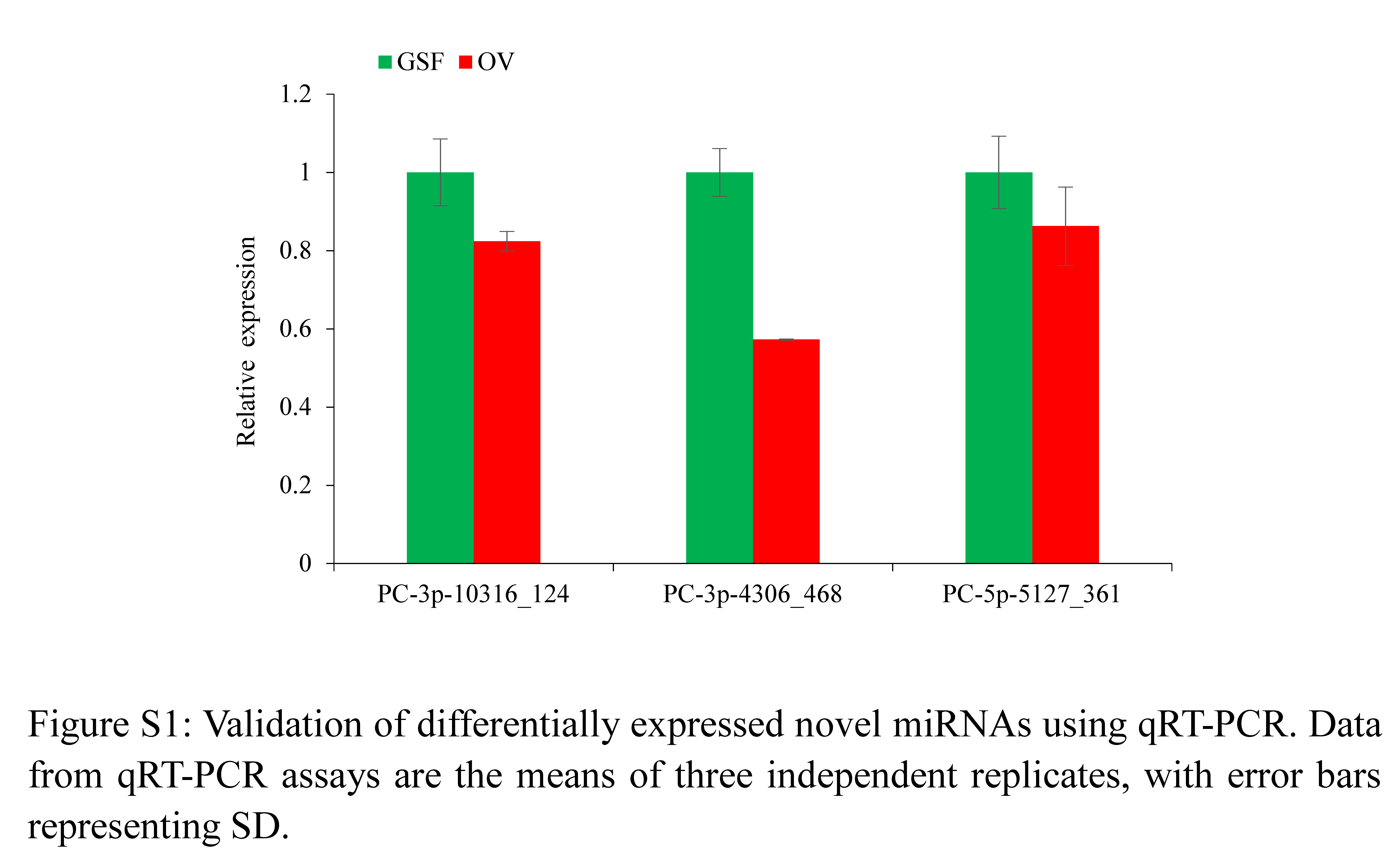

Supplement: Figure S1 [file peerj-07-6267-s001.png]

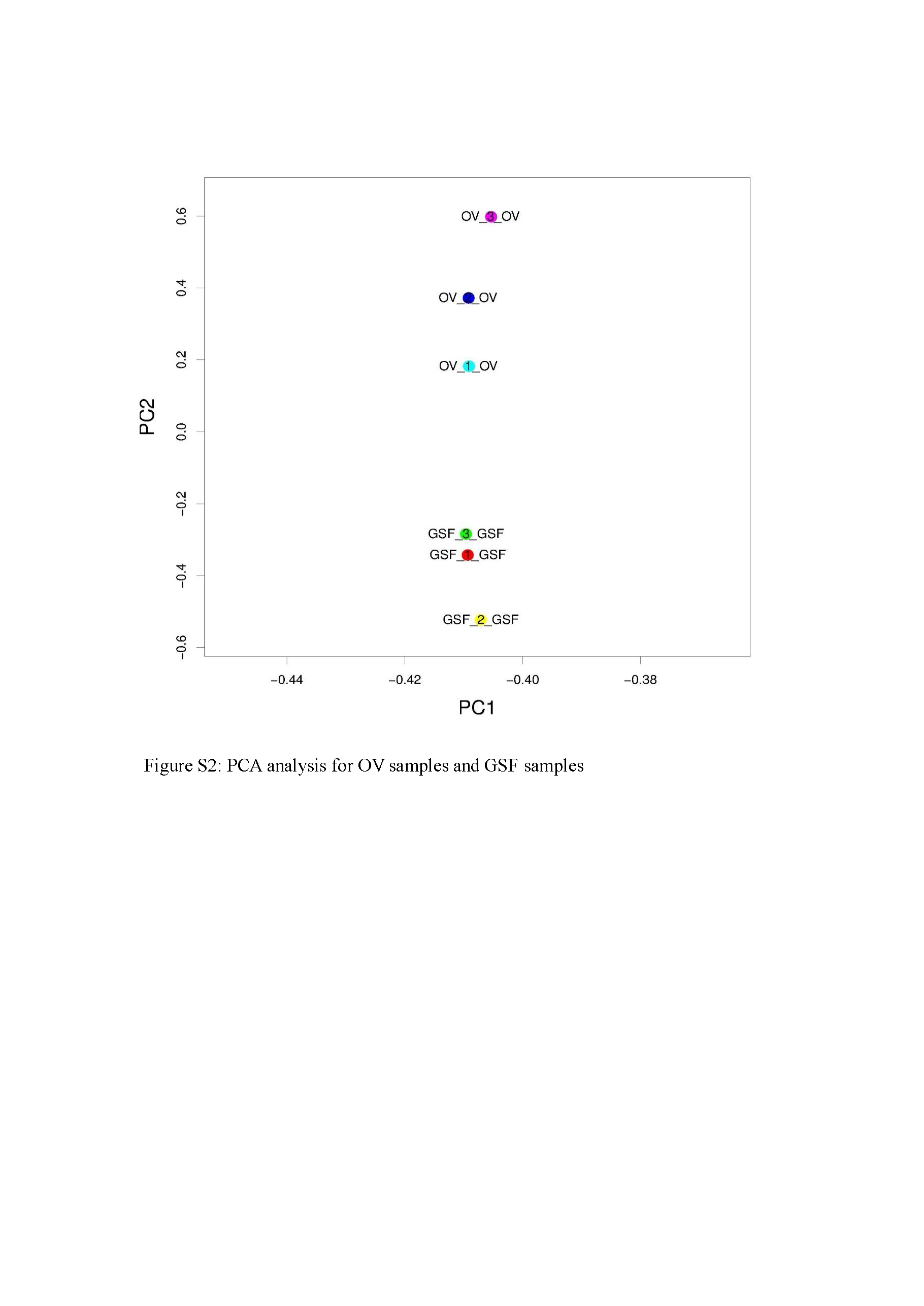

Supplement: Figure S2 [file peerj-07-6267-s002.png]
