# Supplementary material for: Genome-wide analysis of circular RNAs in goat skin fibroblast cells in response to Orf virus infection
Source: PeerJ. 2019 Jan 22;7:e6267. doi: 10.7717/peerj.6267 (PMC6346991; doi:10.7717/peerj.6267)
Supplement: Table S1 [file peerj-07-6267-s003.docx]

| **Primer Name** | **Sequence (5'to3')** |
| --- | --- |
| PC-3p-8215_174 stem-loop | GTCGTATCCAGTGCAGGGTCCGAGGTATTCGCACTGGATACGACTTATTC |
| PC-3p-8215_174 F | CGCGAGATACCGCAGCTAG |
| PC-5p-406_14064 stem-loop | GTCGTATCCAGTGCAGGGTCCGAGGTATTCGCACTGGATACGACTCCCAC |
| PC-5p-406_14064 F | GGAGGGTTTGGGTTTGGTC |
| PC-5p-5127_361 stem-loop | GTCGTATCCAGTGCAGGGTCCGAGGTATTCGCACTGGATACGACAAAACA |
| PC-5p-5127_361 F | AGAGCCGGGGGCAGGC |
| PC-3p-10316_124 stem-loop | GTCGTATCCAGTGCAGGGTCCGAGGTATTCGCACTGGATACGACTGAGCC |
| PC-3p-10316_124 F | GCGAATGATGGAGGCACAC |
| PC-3p-4306_468 stem-loop | GTCGTATCCAGTGCAGGGTCCGAGGTATTCGCACTGGATACGACCTTTTT |
| PC-3p-4306_468 F | GCGCGAGATTGCACAGG |
| PC-5p-2253_1210 stem-loop | GTCGTATCCAGTGCAGGGTCCGAGGTATTCGCACTGGATACGACGGACAA |
| PC-5p-2253_1210 F | CGCGATAAGTTCATTCGGAG |
| Universal Reverse Primer | AGTGCAGGGTCCGAGGTATT |
| U6 F | CGCTTCGGCAGCACATATACTA |
| U6 R | CGCTTCACGAATTTGCGTGTCA |

**Table S1.** List of stem-loop primers, forward primers and universal reverse primer for qRT-PCR validation of differentially expressed novel miRNAs.
